# Traumatic injury of the left anterior descending coronary artery with fistula to the right ventricular outflow tract postgunshot wound

**DOI:** 10.1016/j.radcr.2024.09.117

**Published:** 2024-09-26

**Authors:** Sofia Zeidan, Kwame Atsina, Shirin Jimenez, Mohammad H. Madani

**Affiliations:** a School of Medicine, University of California, Davis, USA; b Division of Cardiovascular Medicine, Department of Internal Medicine, University of California Davis, Davis, USA; c Department of Radiology, University of California, Davis, USA

**Keywords:** Coronary artery fistula, Computed tomography, Coronary angiography

## Abstract

Coronary artery fistulas (CAF) are rare abnormalities involving a connection between a coronary artery and an adjacent vessel or heart chamber. Here we discuss the case of a 47-year-old male patient who had multiple gunshot wounds (GSWs) to the chest and abdomen, suffering a through and through bullet wound to the heart from the left ventricle (LV) through the left anterior descending (LAD) coronary artery and exiting from the right ventricle (RV). At the time of his hospitalization, he underwent a non-ECG gated trauma CT scan and subsequent cardiac catheterization that showed patient has a CAF between the LAD and RVOT. Roughly 3 years after his injury, the patient had an ECG-gated coronary CT scan showing the CAF is still present. The patient is now experiencing symptoms of heart failure with suspected worsening of shunt flow from the fistula. This case sheds light on CAFs, their presentation and potential complications to raise awareness for clinicians and radiologists.

## Introduction

CAFs are rare coronary abnormalities, present in only 0.1%-0.2% of people, that involve an abnormal connection between a coronary artery and an adjacent vessel or heart chamber. The majority of CAFs are congenital but they can be due to iatrogenic causes such as device implantation or cardiac surgery. They can also occur secondary to traumatic events such as GSWs. Many CAFs are asymptomatic and are often diagnosed incidentally during coronary angiography or noninvasive cardiac imaging. However, due to their low resistance, they lead to higher blood flow through them. While small fistulas can close spontaneously, larger ones may progressively dilate and increase their shunt ratio [1].

CAFs are commonly nonthreatening, however, in cases of severe shunting, CAFs can cause cardiac chamber dilatation and present with symptoms of ischemia. The symptoms patients experience are often secondary to the complications themselves. Complications can include steal syndrome in which blood is shunted through the fistula thereby decreasing distal blood flow to the original artery leading to myocardial ischemia resulting from the imbalance of blood flow to meet the muscle's demand [2]. CAFs can also cause thrombosis, embolisms, volume overload, arrhythmias and even cardiac tamponade if they were to rupture [2]. The risk for thrombosis and increases with patient's age and with the size of the fistula [4].

Approximately 40%-60% of CAFs originate from right coronary artery, while 30%-60% originate from the LAD artery and most terminate in a venous chamber or vessel and very rarely terminate into the LV or pericardium. About 90% of CAFs terminate into the right side of the heart [5]. Thus, they often cause a left to right shunting.

Initial diagnostic exams can include chest radiography and electrocardiography (EKG). These are often not enough to provide a full diagnosis, but they can be helpful in uncovering complications of CAFs such as volume overload or ST-segment changes consistent with ischemic changes respectively [3]. A transthoracic echocardiogram (TTE) is unable to show origin or termination of the fistula. Thus, the gold standard in diagnosing CAFs is coronary catheterization and subsequent coronary angiography. Yet, there is a recent switch from coronary angiography to coronary computed tomography angiography (CTA) since CTAs are not invasive and can have a higher rate of detection of CAFs [3,6].

Lastly, treatment of CAFs is not always necessary and the decision to treat largely depends on the patient's symptomatic status, hemodynamic state, and evidence of ischemic changes. Treatment can include surgical repair and catheterization with the aim of embolizing the fistula. Transcatheter closure of CAFs had similar early effectiveness to surgery [7]. Thus, catheterization is now the preferred method since surgery was found to be associated with a higher rate of fistula reoccurrence [3]. Unfortunately, recanalization of the fistula is possible, therefore follow up with patients is very important [8]. Additionally, a possible complication of transcatheter closure of CAFs is coil migration that is used to occlude the fistula [9].

## Case presentation

Patient is a 47-year-old male with GSW to chest, back, abdomen, left arm that was found by emergency medical services with diminished breath sounds on the right and weak pulses. Upon arriving to the emergency department (ED), he received a massive transfusion, and an emergency thoracotomy was performed. The patient was intubated and then immediately taken to the operating room (OR). In the OR, the thoracotomy was converted into a clamshell thoracotomy. It was found that the patient had cardiac tamponade with significant clots. Patient was also found to have suffered a through-through bullet wound to the heart. The entry site of the penetrating injury was the left ventricle (LV) through the left anterior descending (LAD) coronary artery and the exit site of the injury was the right ventricle through the acute marginal region. The penetrating injury was identified in the proximity of the left anterior descending coronary artery and measured approximately 1-cm in size. Both injury sites were repaired with sutures, carefully ensuring that the coronary arteries do not get damaged. During this surgery, bilateral tube thoracostomy placement and intra-aortic balloon pump (IABP) placement for tenuous hemodynamics and patient underwent another substantial transfusion. He was admitted with an open thoracotomy and abdomen.

The next day, the patient was taken to the OR again to close the pericardium with bovine patches. The sites of penetrating myocardial injury were found to be hemostatic. On the third day, the patient was taken to the OR to remove the IABP. A transthoracic echocardiogram (TTE) was performed this day showed normal LV function and a LV ejection fraction (EF) of 60%.

**Fig. 1:**
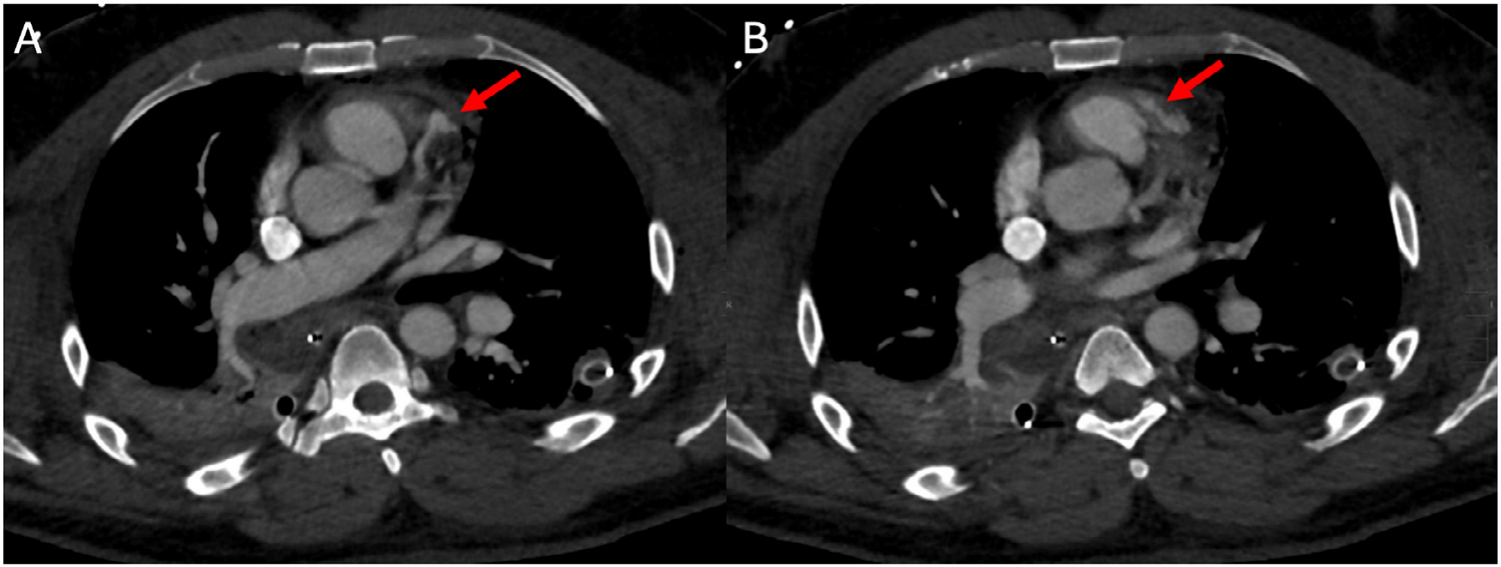
Non-ECG gated contrast enhanced CT scan of the chest. Axial views showing focus of prominent contrast at the left anterior descending coronary artery (LAD) suspicious for traumatic injury (A) with communication to the right ventricular outflow tract (B).

**Fig. 2:**
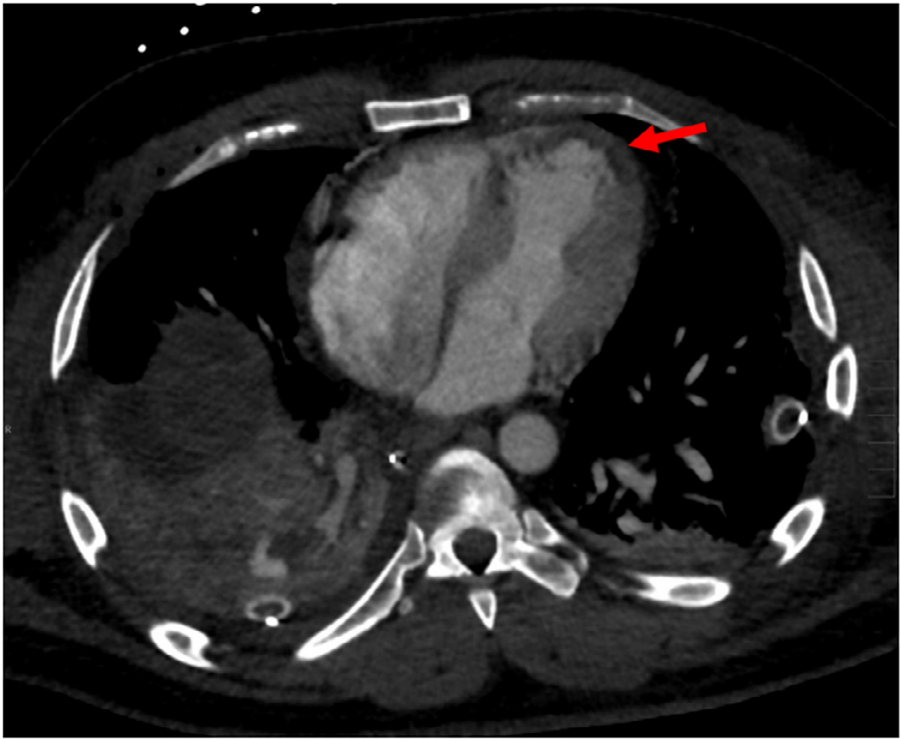
Non-ECG gated contrast enhanced CT scan of the chest. Left ventricular myocardial hypoattenuation suggestive of ischemia/infarct on axial view of the chest.

At this point, the patient became stable enough for a CT scan to evaluate for additional injuries. A non-ECG gated trauma CT scan was performed, and a prominent focal area of contrast was noted in the region of the LAD that appeared to communicate with the RVOT. This finding was reported to be suspicious for traumatic injury with fistulous communication between the LAD and RVOT (Fig. 1). The scan also showed low myocardial hypoattenuation of the left ventricle in the LAD distribution concerning for infarct/ischemia (Fig. 2).

**Fig. 3:**
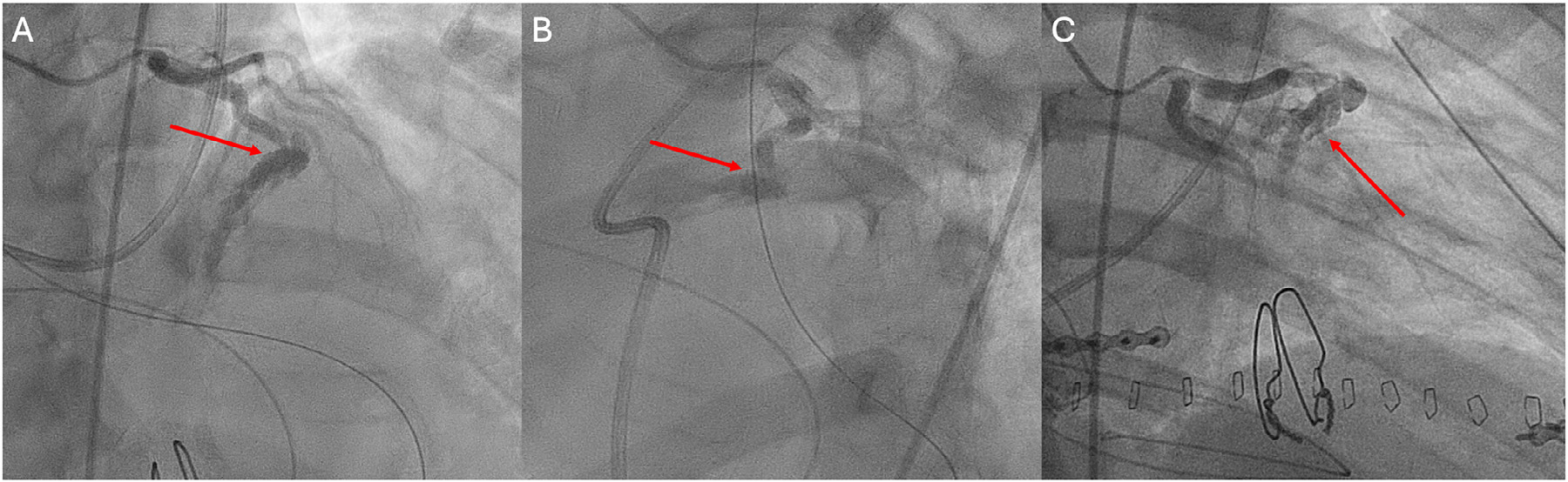
Invasive coronary angiogram. LAD artery in the RAO cranial (A), LAO cranial (B), RAO caudal view (C). Contrast extravasation of the transected left anterior descending artery at its mid segment with fistula formation into the right ventricular outflow tract.

On the same day, the patient developed ST elevations on ECG consistent with STEMI and was taken to the catheterization lab and found to have a transected mid segment of LAD secondary to GSW with fistula formation to the RVOT (Fig. 3). The patient's LAD injury was not considered amenable to percutaneous intervention as it appeared transected. Medical management was performed and the patient was found to have a newly decreased EF (45%) and anteroseptal/apical akinesis on echocardiogram.

A subsequent catheterization a week later was performed to assess the fistula, and found no significant left-to-right shunt with a calculated pulmonary-systemic ratio (Qp:Qs) of 0.96, based on pulse oximetry run. Thus, physicians decided that surgical interventions were not the best choice at this time and continued with the plan of diuresis. Patient was discharged after a 3 month stay to a rehab facility.

**Fig. 4:**
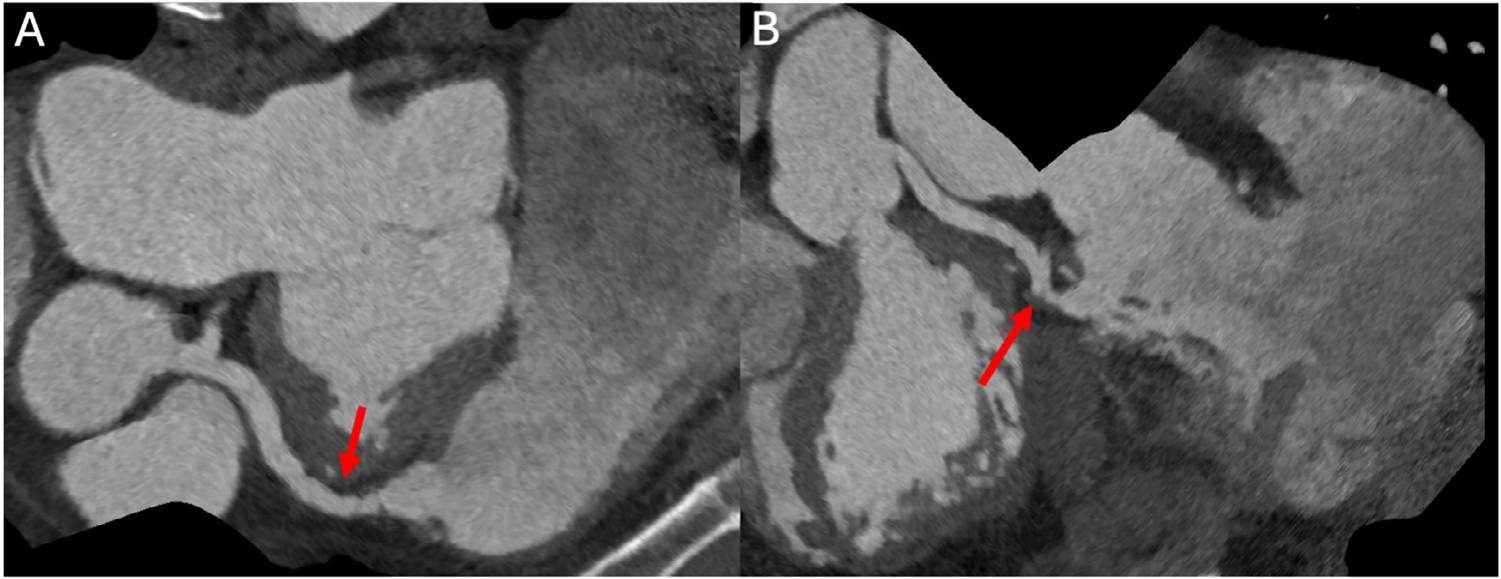
ECG-gated CT coronary angiogram. Curved planar reformations (A, B) demonstrating left anterior descending coronary artery to right ventricular outflow tract fistula.

**Fig. 5:**
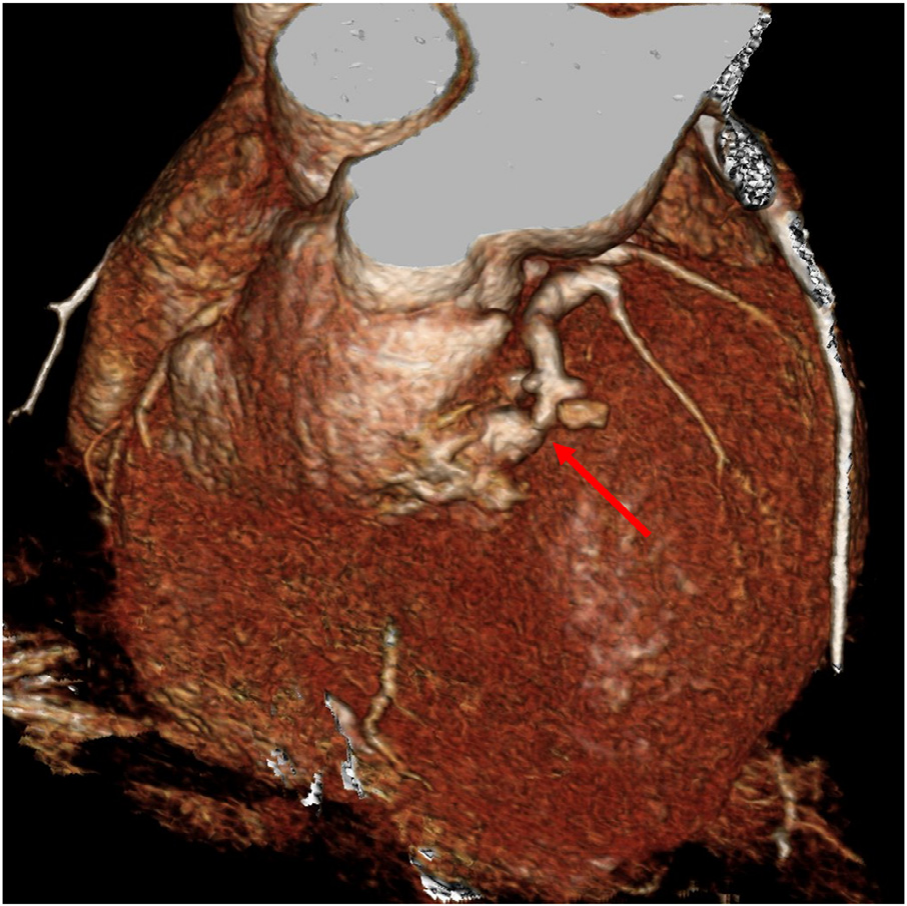
ECG-gated CT coronary angiogram. Volume-rendered 3D reconstruction demonstrating left anterior descending coronary artery to right ventricular outflow tract fistula.

A subsequent ECG-gated coronary CT scan about 3 years after the initial incident shows the fistula is still present. (Figs. 4, 5). Additionally, a TTE completed around the same time showed LV systolic function is moderately reduced with an estimated LV ejection fraction is 35%. At his most recent cardiology visit, the patient endorsed some fatigue after exertion and having to sleep on multiple pillows at night due to shortness of breath. He also states that he has occasional pedal edema. His cardiologist was concerned for a worsening shunt hemodynamics from his fistula and thus the patient was then referred to cardiothoracic surgery. However, the cardiothoracic surgeon stated that there is no bypass option due to his chronically transected LAD. He will not have good conduits due to left and right internal mammary arteries sacrificed during the clamshell thoracotomy done at the time of his injuries. Since then, he has been referred for a left and right heart catheterization to re-evaluate the ratio of the Qp:Qs shunt and to determine if percutaneous coronary artery intervention to occlude the LAD-RVOT fistula is possible.

## Discussion

Coronary artery fistulas (CAFs) are a rare cardiac abnormality that involve an abnormal connection between a coronary artery and an adjacent vessel or heart chamber. Most CAFs are congenital and often found incidentally since most are small and do not have a significant shunt ratio to cause symptoms and can even close spontaneously. Yet, CAFs can enlarge over time and become dangerous. [1].

This case report presents a 47-year-old male patient at the time of his GSW to the chest and the day after his injuries had an ejection fraction that was normal at 65% but worsened to 45% shortly after due to ischemic changes to the heart. He was found to have a CAF from the LAD to the RVOT on CT and subsequent catheterization. Because the gunshot was to the heart, it makes a traumatic etiology of the CAF is much more likely than congenital. The patient had full heart function and no cardiac issues throughout his life, thus a congenital cause of this CAF is highly unlikely. This case highlights the fact that traumatic CAFs can occur post GSW and although the GSW were surgically corrected, a residual CAF remained and contributed if not caused his worsening heart function.

Siebert et al have reported a LAD to RV CAF due to iatrogenic cause following an endomyocardial biopsy. In this patient, he had a heart transplant that required multiple biopsies from the interventricular septum that likely led to his CAF. In comparison with our case, this patient had multiple traumas to the heart muscle via the biopsies, which highlights that a CAF from LAD to RV can be due to biopsies but a single trauma from a GSW in our patient caused severe heart damage showing the danger that CAFs can cause [10]. Moreover, a traumatic stab wound lead to an LAD to RV was reported by Sheikhi et al. [11], however unlike our patient, this patient had underwent repair of the CAF 7 days after admission and he did not end up experiencing worsening heart symptoms which shows the importance of repairing CAF since our patient did not get it repaired and experienced worsening symptoms years down the line. Reyes et al reported a case of a CAF from LAD to RV also due to a traumatic GSW, however here it was recommended that for this patient with a small fistula, surgery is not the best route and conservative management alone was enough [12]. This further emphasizes the need for surgeons and radiologists to work together and decide what is best for each patient based on their own symptoms and workup.

The indications for closing CAFs include closing all large fistulas that have high blood flow and multiple communications or drainage sites with surgery while proximal fistula organs and a single drain site that can be easily accessible are best suited for closure with coils, detachable balloons or vascular plugs [13]. Each patient with a CAF can have a very different presentation from another and so it is important to evaluate each fistula thoroughly while gathering extensive patient symptoms and history to determine the best route for repair or continuous conservative management.

Due to the possible complications that CAFs can create, it is important that providers are aware of them and continue to monitor patients. Roughly 3 years after the patient sustained his injuries, he is developing worsening cardiac symptoms having to sleep on multiple pillows at night due to shortness of breath and experiences dyspnea and fatigue. This is potentially due worsening shunt flow from his coronary fistula. The patient has been referred for cardiac catheterization to measure the shunt ratio of the CAF and potentially embolize the fistula. In addition to not being found as a candidate for surgery due to his discussed LAD, surgery was found to be associated with a higher rate of fistula reoccurrence compared with catheterization [3].

It is very important for a radiologist to report any CAFs seen and for a cardiologist to follow up on patients with CAFs. This condition may result in serious complications. Thus, we aim to shed light on this entity for physicians.

## Conclusion

CAFs involve an abnormal connection between a coronary artery and an adjacent vessel or heart chamber. Patients with CAFs may be asymptomatic or symptomatic. This patient was found to have a CAF from the LAD to the RVOT after a GSW to the heart. We hope to raise awareness of this rare phenomenon for imagers and clinicians.

## Patient consent

Informed and written consent was obtained from the patient for publication of this case report.
